# Integrating overall survival and health-related quality of life to prioritize first-line systemic therapies for unresectable advanced gastric cancer: a network meta-analysis

**DOI:** 10.3389/fonc.2026.1910817

**Published:** 2026-09-28

**Authors:** Ji Wu, Zhigang Zheng, Xiping Shen, Huijuan Dai, Yefeng Wu

**Affiliations:** 1 Department of General surgery, Suzhou Ninth Hospital Affiliated to Soochow University, Suzhou, Jiangsu, China; 2 Department of Interventional Oncology, Renji Hospital, Shanghai Jiao Tong University School of Medicine, Shanghai, China; 3 Department of Breast Surgery, Renji Hospital, Shanghai Jiao Tong University School of Medicine, Shanghai, China

**Keywords:** advanced gastric cancer, health-related quality of life, immunology, network meta-analysis, systemic therapy

## Abstract

**Purpose:**

This study aimed to compare the effects of various first-line systemic therapies on health-related quality of life (HR-QoL) in patients with unresectable advanced gastric cancer (GC), and to comprehensively integrate survival benefits and HR-QoL outcomes associated with different treatments.

**Methods:**

A systematic literature search was conducted in the MEDLINE, CENTRAL and Scopus databases for studies published from database inception to December 2025. Manual retrieval of reference lists and abstracts from major oncology conferences held between 2016 and 2025 was performed as supplementary screening. Eligible studies were phase III randomized controlled trials (RCTs) that compared immune checkpoint inhibitors or targeted agents with platinum-based chemotherapy as first-line regimens for advanced GC and reported data on HR-QoL deterioration. Time to deterioration (TTD) of HR-QoL domains was assessed using the EORTC QLQ-C30, EORTC QLQ-STO22 and EQ-5D questionnaires. The surface under the cumulative ranking curve (SUCRA) was applied to rank all included treatments based on HR-QoL indicators. We further performed an exploratory composite analysis integrating overall-survival and HR-QoL outcomes via the minimum distance criterion.

**Results:**

In total, 10 RCTs enrolling over 8000 patients and covering six HR-QoL domains met the inclusion criteria. SUCRA analyses indicated that trastuzumab and tislelizumab had the lowest probability of the deterioration of global health status, physical function, nausea/vomiting, appetite loss and pain. When HR-QoL was combined with overall survival (OS) via an exploratory minimum distance criterion composite analysis, trastuzumab and tislelizumab showed favorable comprehensive performance across most evaluated domains. This composite metric remains exploratory without established uncertainty estimates and should not serve as primary evidence for clinical decision-making.

**Conclusions:**

This network meta-analysis suggests that trastuzumab (for HER2-positive disease) and tislelizumab (for largely biomarker-unselected populations) deliver the balance between HR-QoL preservation and OS benefit within their respective biomarker-eligible patient subgroups among systemic treatment options for unresectable advanced GC.

**Systematic Review Registration:**

https://www.crd.york.ac.uk/prospero/, identifier CRD420261422326.

## Introduction

Advanced gastric cancer (GC) remains a major global health burden, characterized by high morbidity, poor long-term prognosis and limited curative treatment options (1, 2). The clinical management of advanced GC is further complicated by the coexistence of underlying digestive disorders. They exacerbate disease-related symptoms and impairs patients’ treatment tolerance, ultimately compromising clinical outcomes (3, 4).

The treatment landscape for advanced GC has evolved substantially over the past decades. Platinum-based chemotherapy has long served as the standard first-line regimen (5). In recent years, multiple novel therapeutic modalities have emerged, including combination regimens of immune checkpoint inhibitors (ICIs) plus platinum doublet chemotherapy (e.g., pembrolizumab or nivolumab combined with platinum-based agents) (6, 7). Additionally, biomarker-directed targeted therapies such as anti-HER2 agents, CLDN18.2-targeted drugs and anti-VEGFR monoclonal antibodies combined with chemotherapy have yielded promising efficacy in clinical trials (8–10). While these advances have greatly expanded the armamentarium of first-line treatments for advanced GC, they also pose challenges for clinical decision-making.

Conventional oncology endpoints, namely overall survival (OS) and progression-free survival (PFS), are insufficient to fully reflect the comprehensive clinical value of available regimens (11). Accordingly, health-related quality of life (HR-QoL) has been recognized as an essential complementary endpoint in cancer clinical research (12). This indicator is particularly critical for patients with advanced GC, as both tumor lesions and pre-existing gastrointestinal comorbidities cause a substantial symptomatic burden, which is frequently worsened by systemic therapy. The European Society for Medical Oncology (ESMO) has formally endorsed HR-QoL as a core indicator for evaluating the clinical value of anticancer treatments, and improvements in HR-QoL are proven to elevate the overall assessment of therapeutic efficacy (13).

Despite its well-documented clinical importance, HR-QoL remains understudied and underreported in GC clinical trials. Furthermore, trials adopt heterogeneous assessment tools, ranging from general oncology questionnaires (EORTC QLQ-C30) to GC-specific instruments (EORTC QLQ-STO22) (14, 15). Such methodological heterogeneity hinders direct cross-trial comparisons of HR-QoL outcomes.

Existing network meta-analyses for first-line therapies of advanced GC have primarily focused on survival benefits alone (16–19). However, no study has systematically synthesized HR-QoL data or established a composite evaluation system integrating both survival and HR-QoL profiles.

To address this research gap, we conducted the network meta-analysis to comprehensively compare the effects of different first-line systemic therapies on HR-QoL in patients with advanced GC, using patient-reported outcomes (PROs) as the primary assessment tool. This study also aimed to develop composite endpoints combining survival benefits and HR-QoL changes. By providing a holistic evaluation of therapeutic value, our findings will offer robust evidence to support patient-centered treatment selection in routine clinical practice.

## Methods

This network meta-analysis was performed in compliance with the Preferred Reporting Items for Systematic Reviews and Meta-Analyses (PRISMA) guidelines and prospectively registered on the PROSPERO database (Registration ID: CRD420261422326) (20). Ethical approval and patient informed consent were not required for this analysis, as all data were extracted from published phase III randomized controlled trials (RCTs). All original trials had obtained ethical approval from local institutional review boards and written informed consent from enrolled participants before study initiation.

### Data sources and search strategy

We searched three electronic databases: MEDLINE, Cochrane Central Register of Controlled Trials (CENTRAL) and Scopus, from database inception to December 2025. The complete search strategies are presented in **Supplementary Appendix S1**.

To supplement electronic retrieval, we manually screened reference lists of included original studies and relevant review articles. For trials with incomplete HR-QoL data in the primary full-text publications, we retrieved their *post-hoc* exploratory analyses focused on HR-QoL. For multiple publications from a single RCT, only the most recent and comprehensive dataset was retained to prevent duplicate data extraction.

We also screened abstracts presented at the American Society of Clinical Oncology (ASCO) and ESMO annual conferences between 2016 and 2025. Conference abstracts that were subsequently published as full-text articles already included in this analysis were excluded to avoid data duplication.

### Study selection

Eligible studies were limited to phase III RCTs meeting all the following inclusion criteria: (1) the control group received active treatment; (2) trials were conducted in the first-line treatment setting for advanced GC; (3) trials compared platinum-based chemotherapy with ICIs or targeted agents, including anti-HER2, anti-CLDN18.2 and anti-VEGFR monoclonal antibodies; (4) trials reported time to deterioration (TTD) of HR-QoL scores measured by validated questionnaires; (5) full texts were published in English. Studies were excluded if they combined systemic therapy with locoregional treatments or enrolled patients receiving second-line or subsequent therapy.

Two independent reviewers (Ji Wu, Zhigang Zheng) first screened titles and abstracts, then assessed full texts for final eligibility. Any disagreements between the two reviewers were resolved by group consensus.

### Data extraction and quality assessment

Primary outcomes extracted from eligible trials included the median TTD of each HR-QoL domain, as well as corresponding hazard ratios (HRs) and 95% confidence intervals (95% CIs). HRs for OS were also extracted for the combined analysis of OS and HR-QoL. Notably, the time-to-deterioration of HR-QoL domains is largely driven by treatment-related toxicity and symptom burden rather than purely anti-tumor efficacy mediated by tumor biomarkers.

Predefined study-level covariates were collected, including first author, publication year, intervention and control regimens, types of HR-QoL questionnaires, evaluated HR-QoL domains, and whether HR-QoL analyses were reported in primary publications or *post-hoc* exploratory reports. HR-QoL represents patient-reported subjective experience and can partially reflect treatment tolerability. However, HR-QoL outcomes are not equivalent to investigator-assessed treatment-related adverse events, and cannot serve as a substitute for formal toxicity evaluation. We additionally extracted biomarker eligibility criteria (HER2 status, CLDN18.2 status, PD-L1 selection criteria) for each included trial as study-level covariates. Nevertheless, given that only aggregated published data were available, network meta-regression could not be performed to adjust for inter-trial heterogeneity in biomarker population composition.

The Cochrane Risk of Bias Tool was used to assess the methodological quality of included RCTs (21). Five core domains were evaluated, including random sequence generation, allocation concealment, blinding of participants and investigators, incomplete outcome data and selective outcome reporting. The two independent reviewers completed quality assessment separately, and all disagreements were resolved through consensus.

### Data synthesis and analysis

We conducted Bayesian network meta-analysis to compare TTD across treatment groups, with platinum-based chemotherapy defined as the common reference. Only HR-QoL domains reported in at least four independent RCTs were included in quantitative synthesis. Our evidence network adopts a star-shaped structure without closed loops. There are no available head-to-head RCTs between different experimental agents; all relative comparisons among different treatments are obtained from indirect comparisons against the common platinum-chemotherapy reference, which is accompanied by inherent statistical uncertainty.

Forest plots were used to visualize pairwise comparisons between interventions, and treatment ranking was determined based on HR values. The surface under the cumulative ranking curve (SUCRA) was calculated for each eligible HR-QoL domain (22). SUCRA values range from 0 to 1: a value approaching 1 indicates a higher likelihood of a treatment being ranked first, while a value near 0 corresponds to the lowest ranking.

Frequentist random-effects network meta-analysis was performed for sensitivity analysis to validate the robustness of the Bayesian model (23). P-scores were calculated within the frequentist framework, and this metric represents the mean probability of one intervention being superior to all comparators.

We performed an exploratory composite analysis integrating HR-QoL and overall survival using the minimum distance criterion (12). Briefly, this method computes the Euclidean distance between each treatment’s effect estimates and the hypothetical ideal treatment with optimal performance across both OS and HR-QoL dimensions; smaller distance values indicate better comprehensive performance. This composite approach assigns equal weight to OS and HR-QoL outcomes, assuming patient preferences for survival versus quality-of-life are identical across all individuals, which may not reflect real-world patient priorities. No established methods exist to generate confidence intervals or uncertainty metrics for minimum distance criterion (MDC) point estimates. This approach remains exploratory and has not been broadly validated for oncology settings. Detailed calculation procedures are provided in **Supplementary Appendix S2**.

## Results

### Literature search and study characteristics

The study selection process is presented in **Figure 1**. A total of 415 titles and abstracts were retrieved through the initial literature search. After multi-stage screening, 10 phase III RCTs met the prespecified eligibility criteria and were included in this network meta-analysis (**Supplementary Table 1**).

**Figure 1:**
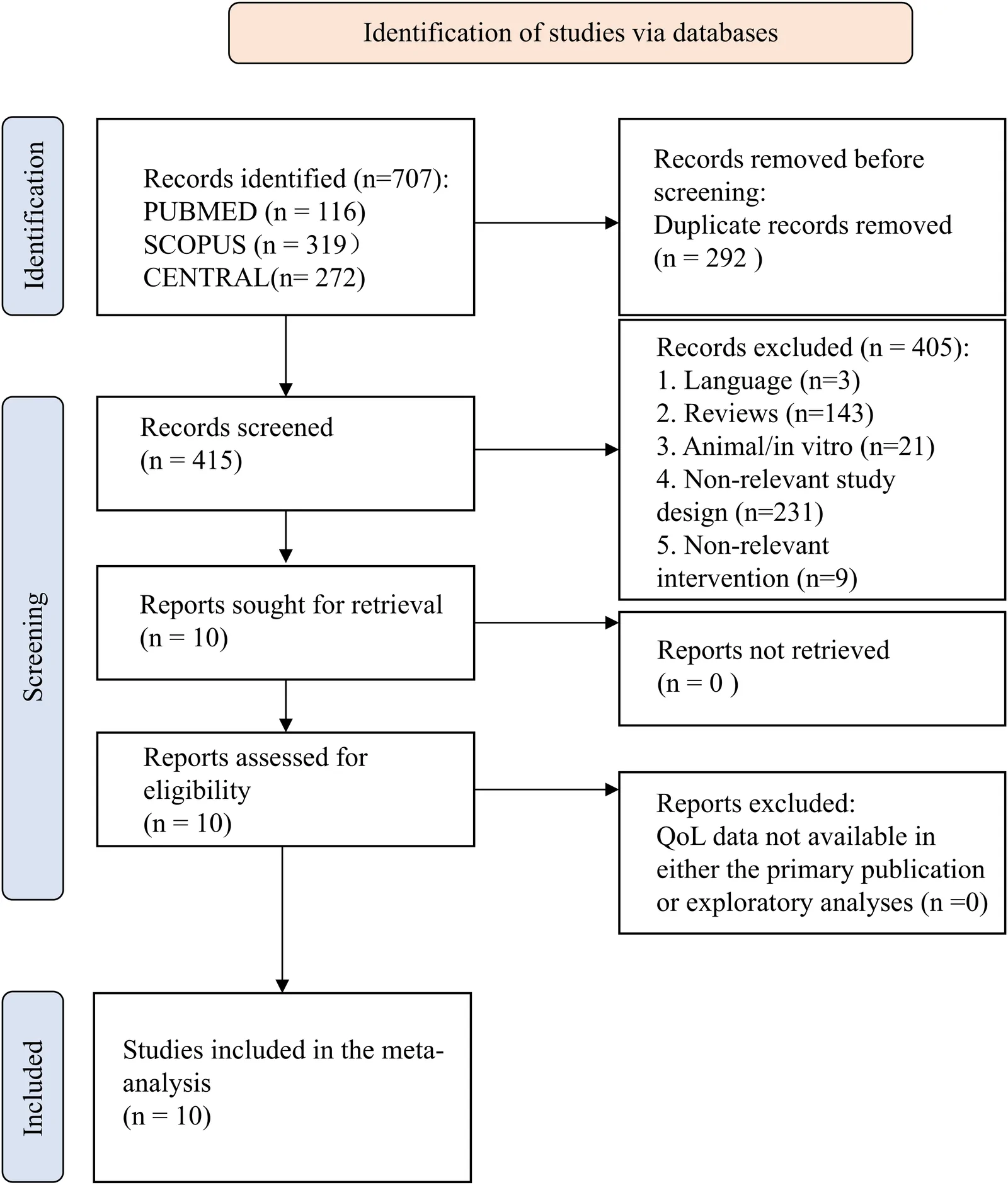
Flow diagram of study selection.

All included RCTs employed a two-group parallel design. Nine trials assigned platinum-based chemotherapy as the control regimen, while one trial used trastuzumab combined with platinum-based chemotherapy in the control arm.

In terms of HR-QoL reporting, six trials published relevant data in their primary full-text articles, and the remaining four reported HR-QoL outcomes in *post-hoc* exploratory analyses. For assessment tools, five trials used both the EORTC QLQ-C30 and EORTC QLQ-STO22; one trial adopted only the EORTC QLQ-C30; one combined the EORTC QLQ-C30 with FACT-Ga; and two paired the EORTC QLQ-C30 with the EORTC QLQ-OG25. Additionally, six trials applied the EQ-5D Utility Scale for evaluation, though detailed quantitative data from this scale were only available in four of them.

In line with the predefined inclusion criteria for outcome metrics, six HR-QoL domains reported in at least four eligible trials were incorporated into quantitative synthesis: global health status/quality of life (GHS/QoL), physical function, nausea/vomiting and appetite loss (assessed via the EORTC QLQ-C30); pain (assessed via the EORTC QLQ-STO22); and Utility Index (UI, assessed via the EQ-5D). Risk of bias assessment revealed an overall low risk of bias across all included trials (**Supplementary Table 2**).

### EORTC-QLQ-C30 domains

GHS/QoL was assessed in eight trials involving eight treatment regimens (**Supplementary Figure 1**). Compared with platinum-based chemotherapy, trastuzumab, tislelizumab and pembrolizumab significantly reduced the risk of GHS/QoL deterioration (**Figure 2A**). Of note, observed inter-treatment differences in HR-QoL deterioration time should be interpreted alongside the known investigator-reported toxicity profile of each regimen, as HR-QoL deterioration may stem from both tumor-related symptoms and treatment-related adverse events. SUCRA analysis showed that trastuzumab yielded the highest probability (89%) of delaying GHS/QoL deterioration (**Table 1**). It should be noted that these trastuzumab-associated results are based solely on patients with HER2-positive advanced gastric cancer.

**Figure 2:**
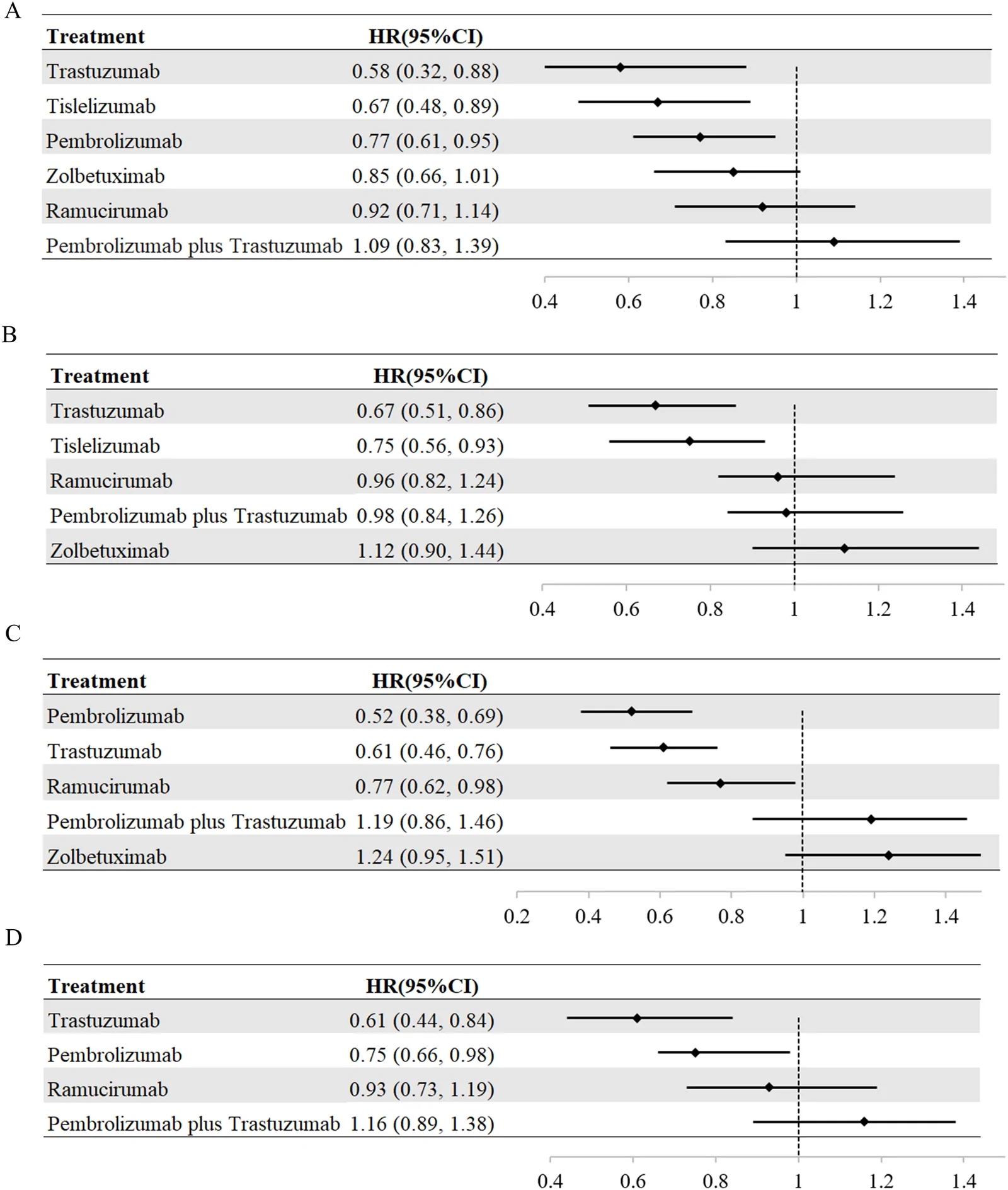
Network meta-analysis of time to deterioration based on EORTC QLQ-C30 domains. **(A)** global health status/quality of life **(B)** physical function **(C)** nausea/vomiting **(D)** appetite loss. Hazard ratio (HR) <1 indicates reduced risk of HR-QoL domain deterioration relative to reference treatment.

**Table 1:** First-line systemic treatments ranked by probability of reducing the risk of deterioration of health-related quality-of-life scores per EORTC-QLQ-C30 responses.

EORTC-QLQ-C30 measure, by treatment	SUCRA
GHS/QoL	
Trastuzumab	0.889
Tislelizumab	0.748
Pembrolizumab	0.560
Zolbetuximab	0.376
chemotherapy	0.360
Ramucirumab	0.346
Pembrolizumab plus Trastuzumab	0.221
Physical functioning	
Trastuzumab	0.856
Tislelizumab	0.815
chemotherapy	0.420
Ramucirumab	0.356
Pembrolizumab plus Trastuzumab	0.352
Zolbetuximab	0.202
Nausea/vomiting	
Pembrolizumab	0.900
Trastuzumab	0.753
Ramucirumab	0.569
chemotherapy	0.444
Pembrolizumab plus Trastuzumab	0.229
Zolbetuximab	0.105
Appetite loss	
Trastuzumab	0.834
Pembrolizumab	0.680
Ramucirumab	0.444
chemotherapy	0.375
Pembrolizumab plus Trastuzumab	0.168

EORTC-QLQ-C30, the European Organization for Research and Treatment of Cancer’s Quality-of-Life Questionnaire Core, 30 items; GHS/QoL, global health status/quality of life; SUCRA, surface under the cumulative ranking.

Trastuzumab results are restricted to HER2-positive patients; zolbetuximab data are limited to CLDN18.2-positive tumours; ICIs include mixed or PD-L1-enriched populations.

Physical function was evaluated across six trials (**Supplementary Figure 2**). Relative to platinum-based chemotherapy, trastuzumab and tislelizumab markedly decreased the risk of deterioration in physical function (**Figure 2B**). Trastuzumab ranked first for this domain, with a SUCRA probability of 86% (**Table 1**).

Six trials reported outcomes for nausea/vomiting (**Supplementary Figure 3**). Pembrolizumab, trastuzumab and ramucirumab demonstrated statistically significant benefits over platinum-based chemotherapy in lowering the risk of symptom deterioration (**Figure 2C**). Pembrolizumab achieved the highest SUCRA probability (90%) for this endpoint (**Table 1**).

Data regarding appetite loss were extracted from four trials (**Supplementary Figure 4**). Trastuzumab and pembrolizumab significantly reduced the risk of appetite loss deterioration versus platinum-based chemotherapy (**Figure 2D**), and trastuzumab yielded the highest SUCRA probability of 83% for this domain (**Table 1**).

Sensitivity analysis using frequentist random-effects network meta-analysis produced results consistent with the primary Bayesian network meta-analysis (**Supplementary Table 3**).

### EORTC QLQ-STO22 domains

Pain severity was evaluated in five trials (**Supplementary Figure 5**). Trastuzumab and tislelizumab significantly reduced the risk of pain deterioration versus platinum-based chemotherapy (**Figure 3A**). Trastuzumab achieved the highest SUCRA probability of 76% for this domain (**Table 2**). The frequentist network meta-analysis for sensitivity analysis confirmed the robustness of the above results (**Supplementary Table 4**).

**Figure 3:**
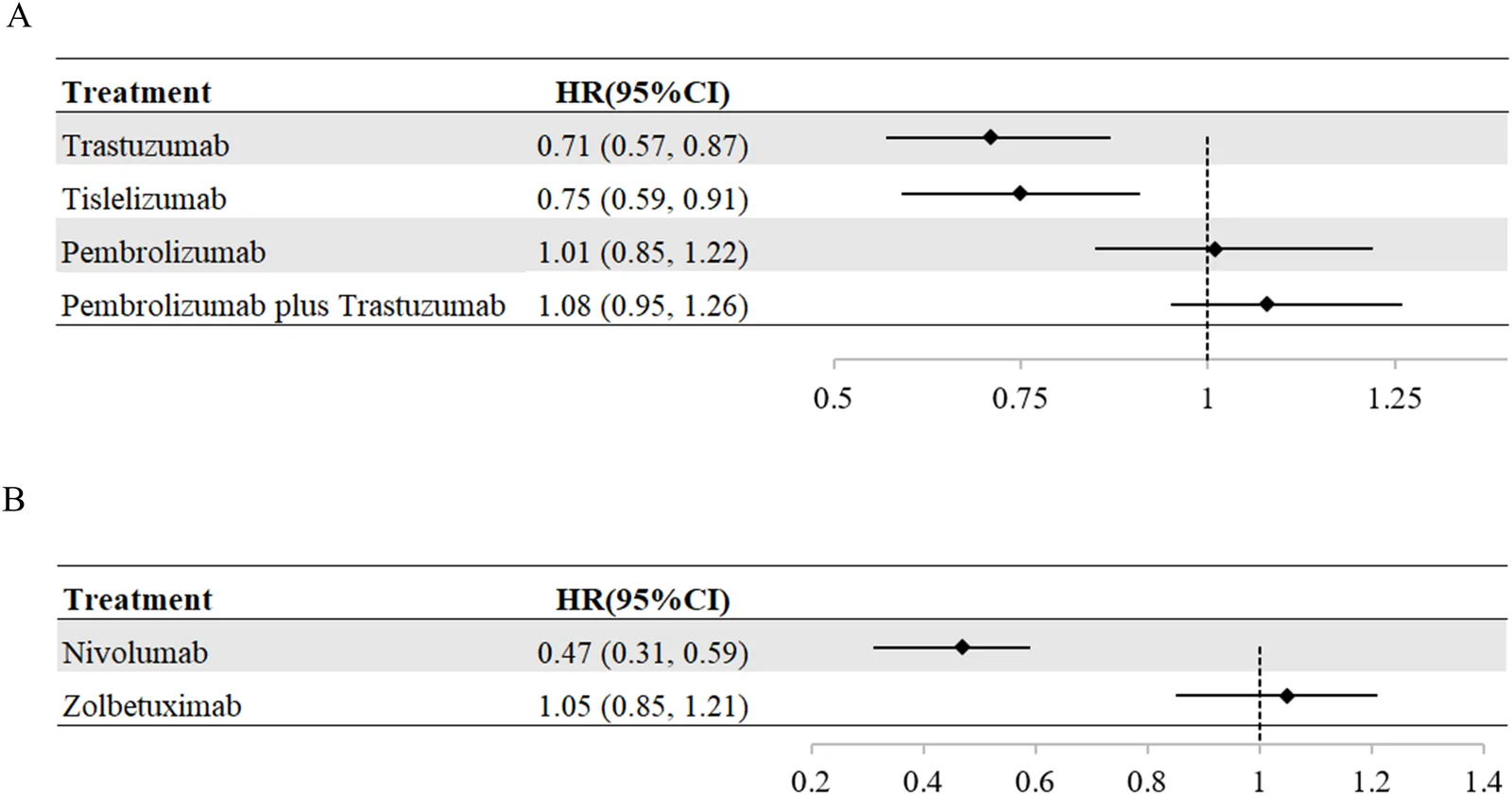
Network meta-analysis of time to deterioration based on EORTC QLQ-STO22 and EQ-5D domains. **(A)** pain **(B)** utility index. Hazard ratio (HR) <1 indicates reduced risk of HR-QoL domain deterioration relative to reference treatment.

**Table 2:** First-line systemic treatments ranked by probability of reducing the risk of deterioration of health-related quality-of-life scores per EORTC-QLQ-STO22/EQ-5D responses.

EORTC-QLQ- STO22 measure, by treatment	SUCRA
Pain	
Trastuzumab	0.759
Tislelizumab	0.737
Pembrolizumab	0.449
Pembrolizumab plus Trastuzumab	0.325
chemotherapy	0.230
EQ-5D measure, by treatment	
Utility index	
Nivolumab	0.948
chemotherapy	0.365
Zolbetuximab	0.187

EORTC-QLQ-STO22, the EORTC Quality of Life Questionnaire – Gastric Cancer Module; EQ-5D, EuroQol Five-Dimensional Questionnaire; SUCRA, surface under the cumulative ranking.

Trastuzumab results are restricted to HER2-positive patients; zolbetuximab data are limited to CLDN18.2-positive tumours; ICIs include mixed or PD-L1-enriched populations.

### EQ-5D domains

UI data were available from four trials (**Supplementary Figure 6**). Nivolumab was associated with a significantly lower risk of UI deterioration compared with platinum-based chemotherapy (**Figure 3B**), with the highest SUCRA probability of 95% across all interventions (**Table 2**).

### OS and HR-QoL

SUCRA values from the network meta-analysis of OS are presented in **Supplementary Table 5**. MDC values for the combined evaluation of HR-QoL domains and OS are summarized in **Supplementary Table 6**. Overall, trastuzumab exhibited the most favorable point-estimate performance across most HR-QoL domains in this exploratory MDC-based integrated analysis. No uncertainty metrics are available for these composite rankings. The MDC values ranged from 0.287 (for GHS/QoL) to 0.362 (for nausea/vomiting), both derived from EORTC QLQ-C30 assessments.

### Sensitivity analysis

An additional threat to transitivity arises from inconsistent control-arm design: one included trial (KEYNOTE-811) used trastuzumab-plus-chemotherapy rather than platinum-chemotherapy alone as its control group. Four of the included trials reported HR-QoL outcomes from post-hoc exploratory analyses, which may introduce reporting bias. We therefore performed a prespecified sensitivity network meta-analysis excluding these four post-hoc-based datasets; treatment rankings from this sensitivity analysis were broadly consistent with our primary analysis (**Supplementary Tables 7**-**10**). Treatment rankings remained broadly consistent with primary analysis after excluding post-hoc HR-QoL datasets, and after restricting analysis to trials applying ≥10-point TTD deterioration threshold. Only minor rank fluctuations were observed across individual HR-QoL domains without altering our overall inference. We also conducted sensitivity analyses with alternative weighting schemes for OS versus HR-QoL(**Supplementary Table 11**).

## Discussion

PROs have become essential endpoints in modern oncology research, as they enable patient-centered assessment of treatment efficacy and provide prognostic information beyond conventional survival metrics (24). Assessing HR-QoL poses particular challenges for patients with advanced GC, due to overlapping symptomatic burdens caused by tumor lesions and pre-existing chronic gastrointestinal comorbidities. While the therapeutic armamentarium for advanced GC has expanded considerably in recent years, most existing comparative studies have focused solely on survival endpoints (25–27). Few studies have comprehensively compared PROs and survival benefits across ICIs and targeted agents, which limits a full understanding of the net clinical benefit of currently approved phase III regimens.

The present network meta-analysis included 10 phase III RCTs involving over 8,000 patients with unresectable advanced GC. We addressed this key research gap by integrating survival outcomes and multidimensional HR-QoL data to compare first-line systemic therapies. A combination of general oncology questionnaires (EORTC QLQ-C30, EQ-5D) and GC-specific tools (EORTC QLQ-STO22) was used to comprehensively profile patients’ HR-QoL. The pooled results indicated that trastuzumab and tislelizumab delivered favorable efficacy across most evaluated HR-QoL domains, achieving a balance between long-term HR-QoL preservation and OS benefits relative to other comparators (28, 29).

Stratified analyses across six HR-QoL domains further revealed distinct advantages among different treatment regimens. Trastuzumab and tislelizumab exerted superior efficacy in delaying deterioration in global health status, physical function, appetite loss and pain. In contrast, pembrolizumab showed the strongest efficacy for relieving nausea and vomiting, while nivolumab yielded optimal results on the EQ-5D UI (30–32). The use of multiple validated assessment tools enabled a detailed evaluation of treatment effects on specific patient-reported symptoms, an aspect often overlooked in conventional efficacy assessments. Notably, trastuzumab and tislelizumab not only preserved general quality of life domains common to all cancer patients but also produced marked improvements in GC-specific symptoms such as upper abdominal pain. Their consistent benefits across both universal and disease-specific HR-QoL domains reflect their strong capacity to alleviate the complex, multifaceted symptom burden associated with advanced GC.

The divergent performance across HR-QoL domains among included regimens can be further explained by their pharmacological characteristics and safety profiles. It should be emphasized that mechanistic interpretations presented below draw on external pre-clinical and clinical evidence and remain hypothetical; these causal mechanisms cannot be directly validated by outcomes from the present network meta-analysis. It should also be emphasized that HR-QoL reflects patient-perceived symptom burden, which is related but not identical to investigator-graded adverse events. Some objectively recorded adverse events may cause little subjective distress for patients, whereas meaningful HR-QoL worsening may occur even in the absence of high-grade documented toxicities. As a human epidermal growth factor receptor 2 (HER2)-targeted monoclonal antibody, trastuzumab is hypothesized to have low inherent toxicity. Its most common adverse events are mild, asymptomatic gastrointestinal discomfort and fatigue (33, 34). Rigorous pre-treatment patient selection substantially reduces the incidence of severe complications such as cardiotoxicity, which could potentially account for the minimal physical distress observed among recipients in relevant trials. Tislelizumab has a structurally optimized Fc region, a feature from pre-clinical studies that differentiates it from other programmed death-1 (PD-1) inhibitors including pembrolizumab (35). This structural modification may potentially reduce the incidence and severity of acute immune-related adverse events (irAEs) (25). Fewer immune-related toxicities, together with its favorable tolerability profile, may collectively help sustain patients’ daily function and delay HR-QoL deterioration during treatment.

The combination of pembrolizumab plus trastuzumab has achieved promising survival outcomes in the KEYNOTE-811 trial, leading to its regulatory approval as a first-line regimen for advanced GC (36). Although trastuzumab monotherapy presents favorable tolerability, the combination of pembrolizumab and trastuzumab may bring superimposed immune-related toxicities. Cumulative chronic irAEs and long treatment duration might worsen patients’ symptoms and compromise HR-QoL (37). In addition to ICIs and anti-HER2 agents, CLDN18.2-targeted zolbetuximab also showed suboptimal performance in HR-QoL outcomes in our analysis (38–40). Although the overall incidence of TRAEs was comparable between the pembrolizumab-trastuzumab combination and zolbetuximab, their distinct toxicity spectra may result in different impacts on HR-QoL. Adverse events associated with trastuzumab monotherapy are mostly asymptomatic, and severe toxicities are rare when patients are appropriately selected. In contrast, both the pembrolizumab-trastuzumab combination and zolbetuximab carry a higher risk of unpredictable irAEs. Subsequent worsening of symptoms and the need for corticosteroid intervention might ultimately offset the HR-QoL improvements observed in treated patients.

### Strengths of the study

This analysis has several notable methodological and clinical strengths. Previous network meta-analyses on first-line therapies for advanced GC evaluated survival outcomes and safety separately while largely ignoring patient-reported outcomes (PROs). Following the value framework proposed by the American Society of Clinical Oncology (ASCO), which recommends integrating survival benefits and toxicity to quantify net health benefit for shared clinical decision-making, we conducted an exploratory combined assessment of OS and HR-QoL. This integrated evaluation of net therapeutic benefit represents a clear advancement over earlier analytical models that separated survival endpoints from PROs. The favorable survival and HR-QoL profiles of trastuzumab and tislelizumab provide evidence to support their use as the first-line options for their corresponding biomarker-defined patient populations with unresectable advanced GC. It also confirms that substantial survival gains can be achieved alongside sustained quality of life in this patient population.

### Limitations and future perspectives

Several methodological limitations of this study should be acknowledged. First, HR-QoL data for four included trials originated from post-hoc exploratory analyses instead of prospectively defined trial endpoints. Post-hoc patient-reported outcome analyses are susceptible to reporting bias and multiple-testing inflation; accordingly, HR-QoL results from these trials should be interpreted cautiously. We did not perform quantitative synthesis of investigator-reported objective treatment-related adverse events. All inferences regarding treatment tolerability are derived exclusively from patient-reported HR-QoL metrics; HR-QoL cannot substitute formal comprehensive toxicity evaluation.

Second, our exploratory analysis on MDC used to integrate survival and QoL outcomes assumes independence between these parameters, which may not fully reflect their complex relationship in clinical practice, and it cannot provide measures of uncertainty or variability around the MDC values, which limits robust statistical comparison between treatments. Besides, it should be noted that SUCRA reflects the probability of a treatment being top-ranked in network comparisons and does not directly reflect the magnitude of clinical difference between competing regimens. Differences in SUCRA values do not necessarily correspond to clinically meaningful treatment effects.

Third, clinical trials adopted variable operational definitions for TTD, with inconsistent threshold values for defining score deterioration and variable requirements for confirming sustained symptom worsening. Although we restricted pooled analyses to HR-QoL domains reported in at least four RCTs and adopted random-effects models for analysis, random-effects approaches can only handle statistical heterogeneity rather than fully resolve measurement-driven methodological heterogeneity. TTD endpoints are subject to censoring from disease progression and patient death. Deterioration of HR-QoL scores may result from tumor progression itself rather than treatment-associated toxicities. Therefore, TTD cannot purely reflect treatment tolerability, and this form of censoring may bring potential bias to our HR-QoL estimates.

Fourth, the included trials enrolled heterogeneous biomarker-defined patient populations, including HER2-positive, CLDN18.2-positive, PD-L1-selected, and unselected HER2-negative patients. Differences in biomarker eligibility across studies pose a potential threat to the transitivity assumption of network meta-analysis. Notably, trastuzumab data were exclusively derived from HER2-positive patients, zolbetuximab data were restricted to CLDN18.2-positive tumors, whereas other immune checkpoint inhibitor trials enrolled mixed or PD-L1-enriched populations. Since we only had access to aggregate data, we could not conduct network meta-regression to account for these inter-trial differences. Individual-patient-data network meta-analysis would be required to properly adjust for such population imbalances.

Overall, this network meta-analysis provides comprehensive evidence for clinical treatment selection from the perspectives of therapeutic efficacy and patient experience. The integrated evaluation framework combining survival and HR-QoL also serves as a valuable reference for outcome assessment in future GC clinical trials and real-world studies. In particular, rankings derived from the MDC composite metric are exploratory, lack uncertainty quantification, and should not be used as primary evidence guiding clinical treatment choices.

## Conclusion

This network meta-analysis provides evidence that ICIs and targeted agents achieve the balance between survival gains and sustained HR-QoL within their respective biomarker-eligible populations. Specifically, trastuzumab shows this favorable profile for HER2-positive unresectable advanced GC, while tislelizumab demonstrates balanced outcomes in largely biomarker-unselected patients. Composite endpoints combining survival and HR-QoL constitute a more patient-centered evaluation framework, yet treatment rankings must be interpreted with consideration of trial-level differences in biomarker enrolment criteria and cannot be generalized across distinct biomarker subgroups.

## Data Availability

The original contributions presented in the study are included in the article/**Supplementary Material**. Further inquiries can be directed to the corresponding author.

## References

[1] Duan Y Xu Y Dou Y Xu D . Helicobacter pylori and gastric cancer: mechanisms and new perspectives. J Hematol Oncol. (2025) 18:10. doi: 10.1186/s13045-024-01654-2 39849657 PMC11756206

[2] Sundar R Nakayama I Markar SR Shitara K van Laarhoven HWM Janjigian YY . Gastric cancer. Lancet. (2025) 405:2087–102. doi: 10.1016/s0140-6736(25)00052-2 40319897

[3] Guan WL He Y Xu RH . Gastric cancer treatment: recent progress and future perspectives. J Hematol Oncol. (2023) 16:57. doi: 10.1186/s13045-023-01451-3 37245017 PMC10225110

[4] Jin X Liu Z Yang D Yin K Chang X . Recent progress and future perspectives of immunotherapy in advanced gastric cancer. Front Immunol. (2022) 13:948647. doi: 10.3389/fimmu.2022.948647 35844558 PMC9284215

[5] Kim R An M Lee H Mehta A Heo YJ Kim KM . Early tumor-immune microenvironmental remodeling and response to first-line fluoropyrimidine and platinum chemotherapy in advanced gastric cancer. Cancer Discov. (2022) 12:984–1001. doi: 10.1158/2159-8290.Cd-21-0888 34933901 PMC9387589

[6] Janjigian YY Ajani JA Moehler M Shen L Garrido M Gallardo C . First-line nivolumab plus chemotherapy for advanced gastric, gastroesophageal junction, and esophageal adenocarcinoma: 3-year follow-up of the phase III CheckMate 649 trial. J Clin Oncol Off J Am Soc Clin Oncol. (2024) 42:2012–20. doi: 10.1200/jco.23.01601 38382001 PMC11185916

[7] Shitara K Rha SY Wyrwicz LS Oshima T Karaseva N Osipov M . Neoadjuvant and adjuvant pembrolizumab plus chemotherapy in locally advanced gastric or gastro-oesophageal cancer (KEYNOTE-585): an interim analysis of the multicentre, double-blind, randomised phase 3 study. Lancet Oncol. (2024) 25:212–24. doi: 10.1016/s1470-2045(23)00541-7 38134948

[8] Chen X Xu H Chen X Xu T Tian Y Wang D . First-line camrelizumab (a PD-1 inhibitor) plus apatinib (an VEGFR-2 inhibitor) and chemotherapy for advanced gastric cancer (SPACE): a phase 1 study. Signal Transduction Targeted Ther. (2024) 9:73. doi: 10.1038/s41392-024-01773-9 38528050 PMC10963362

[9] Hu HH Wang SQ Zhao H Chen ZS Shi X Chen XB . HER2(+) advanced gastric cancer: Current state and opportunities (Review). Int J Oncol. (2024) 64:5624–33. doi: 10.3892/ijo.2024.5624 38391024 PMC10901538

[10] Shah MA Shitara K Ajani JA Bang YJ Enzinger P Ilson D . Zolbetuximab plus CAPOX in CLDN18.2-positive gastric or gastroesophageal junction adenocarcinoma: the randomized, phase 3 GLOW trial. Nat Med. (2023) 29:2133–41. doi: 10.1038/s41591-023-02465-7 37524953 PMC10427418

[11] Patel AK Sethi NS Park H . Gastric cancer: A review. Jama. (2026) 335:439–50. doi: 10.1001/jama.2025.20034 41499132

[12] Celsa C Di Maria G Lombardi P D'Alessio A Fulgenzi CAM Brunetti L . Integrating quality of life and survival in systemic therapy for advanced hepatocellular carcinoma: A network meta-analysis. JAMA Oncol. (2025) 11:1160–8. doi: 10.1001/jamaoncol.2025.2470 40810932 PMC12355390

[13] Cherny NI Sullivan R Dafni U Kerst JM Sobrero A Zielinski C . A standardised, generic, validated approach to stratify the magnitude of clinical benefit that can be anticipated from anti-cancer therapies: the European Society for Medical Oncology Magnitude of Clinical Benefit Scale (ESMO-MCBS). Ann Oncol Off J Eur Soc For Med Oncol. (2017) 28:2901–5. doi: 10.1093/annonc/mdw258 27604385

[14] Eom BW Yoon HM Kim YW Min JS An JY Hur H . Quality of life and nutritional outcomes of stomach-preserving surgery for early gastric cancer: A secondary analysis of the SENORITA randomized clinical trial. JAMA Surg. (2024) 159:900–8. doi: 10.1001/jamasurg.2024.1210 38809537 PMC11137655

[15] Lowery MA Wyrwicz LS Oh DY Shiu KK Yañez P Bai Y . First-line pembrolizumab plus chemotherapy for advanced HER2-negative gastric or gastroesophageal junction adenocarcinoma: Health-related quality of life from the randomized phase III KEYNOTE-859 trial. Eur J Cancer (Oxford Engl 1990). (2025) 231:115807. doi: 10.1016/j.ejca.2025.115807 41175805

[16] Kong M Chen H Zhang R Sheng H Li L . Overall survival advantage of omentum preservation over omentectomy for advanced gastric cancer: A systematic review and meta-analysis. World J Surg. (2022) 46:1952–61. doi: 10.1007/s00268-022-06562-5 35462593

[17] Lin Y Liao Y Shen J . First-line immune checkpoint inhibitors with chemotherapy in advanced gastric and gastroesophageal junction adenocarcinoma: a meta-analysis of phase 3 trials. Front Immunol. (2025) 16:1564604. doi: 10.3389/fimmu.2025.1564604 40386769 PMC12081356

[18] Pu W Li S Zhang J Huang J Li J Jiang Y . The efficacy and safety of PD-1/PD-L1 inhibitors in combination with chemotherapy as a first-line treatment for unresectable, locally advanced, HER2-negative gastric or gastroesophageal junction cancer: a meta-analysis of randomized controlled trials. Front Immunol. (2025) 16:1566939. doi: 10.3389/fimmu.2025.1566939 40207218 PMC11979168

[19] Zhang Y Peng W Yang W Zhang W Fan Y . Efficacy and safety of programmed cell death protein-1 inhibitor for first-line therapy of advanced gastric or gastroesophageal junction cancer: a network meta-analysis. Front Immunol. (2025) 16:1500954. doi: 10.3389/fimmu.2025.1500954 40264761 PMC12011870

[20] Hutton B Salanti G Caldwell DM Chaimani A Schmid CH Cameron C . The PRISMA extension statement for reporting of systematic reviews incorporating network meta-analyses of health care interventions: checklist and explanations. Ann Intern Med. (2015) 162:777–84. doi: 10.7326/m14-2385 26030634

[21] Higgins JP Altman DG Gøtzsche PC Jüni P Moher D Oxman AD . The Cochrane Collaboration's tool for assessing risk of bias in randomised trials. BMJ. (2011) 343:d5928. doi: 10.1136/bmj.d5928 22008217 PMC3196245

[22] Owen PJ Miller CT Mundell NL Verswijveren S Tagliaferri SD Brisby H . Which specific modes of exercise training are most effective for treating low back pain? Network meta-analysis. Br J Sports Med. (2020) 54:1279–87. doi: 10.1136/bjsports-2019-100886 31666220 PMC7588406

[23] Pillinger T Arumuham A McCutcheon RA D'Ambrosio E Basdanis G Branco M . The effects of antidepressants on cardiometabolic and other physiological parameters: a systematic review and network meta-analysis. Lancet. (2025) 406:2063–77. doi: 10.1016/s0140-6736(25)01293-0 41135546

[24] Lee MK Oh J . Patient-reported outcomes of regular aerobic exercise in gastric cancer. Cancers. (2021) 13:2080–91. doi: 10.3390/cancers13092080 33923080 PMC8123334

[25] Ajani JA Alsina M Moehler M Lee KW Tang W Steenkamp J . Comparative efficacy and safety of tislelizumab and other programmed cell death protein 1 inhibitors in first-line treatment of advanced gastroesophageal cancers: a systematic review and network meta-analysis. Gastric Cancer. (2025) 28:1021–32. doi: 10.1007/s10120-025-01660-4 41045401 PMC12630173

[26] Liu S Wong HY Xie L Kim Y Shu D Zheng B . Comparative efficacy and tolerability of targeted and immunotherapy combined with chemotherapy as first-line treatment for advanced gastric cancer: a Bayesian network meta-analysis. Sci Rep. (2022) 12:22024. doi: 10.1038/s41598-022-24426-9 36539429 PMC9768117

[27] Zhang W Guo K Zheng S . Immunotherapy combined with chemotherapy in the first-line treatment of advanced gastric cancer: systematic review and Bayesian network meta-analysis based on specific PD-L1 CPS. Curr Oncol (Toronto Ont). (2025) 32:112–25. doi: 10.3390/curroncol32020112 39996912 PMC11854702

[28] Cruz-Correa M Oh DY Kato K Tabernero J Bai Y Shi J . Tislelizumab + chemotherapy in gastric cancer: long-term RATIONALE-305 randomized trial follow-up. Adv Ther. (2026) 43:165–83. doi: 10.1007/s12325-025-03415-0 41251890 PMC12858542

[29] Satoh T Bang YJ Gotovkin EA Hamamoto Y Kang YK Moiseyenko VM . Quality of life in the trastuzumab for gastric cancer trial. Oncologist. (2014) 19:712–9. doi: 10.1634/theoncologist.2014-0058 24951609 PMC4077451

[30] Kang YK Chen LT Ryu MH Oh DY Oh SC Chung HC . Nivolumab plus chemotherapy versus placebo plus chemotherapy in patients with HER2-negative, untreated, unresectable advanced or recurrent gastric or gastro-oesophageal junction cancer (ATTRACTION-4): a randomised, multicentre, double-blind, placebo-controlled, phase 3 trial. Lancet Oncol. (2022) 23:234–47. doi: 10.1016/s1470-2045(21)00692-6 35030335

[31] Moehler M Xiao H Blum SI Elimova E Cella D Shitara K . Health-related quality of life with nivolumab plus chemotherapy versus chemotherapy in patients with advanced gastric/gastroesophageal junction cancer or esophageal adenocarcinoma from CheckMate 649. J Clin Oncol Off J Am Soc Clin Oncol. (2023) 41:5388–99. doi: 10.1200/jco.23.00170 37713657 PMC10713185

[32] Van Cutsem E Valderrama A Bang YJ Fuchs CS Shitara K Janjigian YY . Quality of life with first-line pembrolizumab for PD-L1-positive advanced gastric/gastroesophageal junction adenocarcinoma: results from the randomised phase III KEYNOTE-062 study. ESMO Open. (2021) 6:100189. doi: 10.1016/j.esmoop.2021.100189 34371381 PMC8358416

[33] Bang YJ Van Cutsem E Feyereislova A Chung HC Shen L Sawaki A . Trastuzumab in combination with chemotherapy versus chemotherapy alone for treatment of HER2-positive advanced gastric or gastro-oesophageal junction cancer (ToGA): a phase 3, open-label, randomised controlled trial. Lancet. (2010) 376:687–97. doi: 10.1016/s0140-6736(10)61121-x 20728210

[34] Gunturu KS Woo Y Beaubier N Remotti HE Saif MW . Gastric cancer and trastuzumab: first biologic therapy in gastric cancer. Ther Adv Med Oncol. (2013) 5:143–51. doi: 10.1177/1758834012469429 23450234 PMC3556876

[35] Zhang L Geng Z Hao B Geng Q . Tislelizumab: a modified anti-tumor programmed death receptor 1 antibody. Cancer Control J Moffitt Cancer Center. (2022) 29:10732748221111296. doi: 10.1177/10732748221111296 35926155 PMC9358212

[36] Janjigian YY Kawazoe A Xu J Lonardi S Metges JP Wyrwicz LS . Pembrolizumab with trastuzumab and chemotherapy for HER2-positive advanced gastric cancer: health-related quality-of-life analysis from the randomized KEYNOTE-811 trial. ESMO Open. (2025) 10:105542. doi: 10.1016/j.esmoop.2025.105542 41033280 PMC12514504

[37] Barron CC Stefanova I Cha Y Elsolh K Zereshkian A Gaafour N . Chronic immune-related adverse events in patients with cancer receiving immune checkpoint inhibitors: a systematic review. J Immunother Cancer. (2023) 11:6500–12. doi: 10.1136/jitc-2022-006500 37536939 PMC10401216

[38] Lordick F Van Cutsem E Shitara K Xu RH Ajani JA Shah MA . Health-related quality of life in patients with CLDN18.2-positive, locally advanced unresectable or metastatic gastric or gastroesophageal junction adenocarcinoma: results from the SPOTLIGHT and GLOW clinical trials. ESMO Open. (2024) 9:103663. doi: 10.1016/j.esmoop.2024.103663 39146670 PMC11374961

[39] Mitsui Y Sato Y Shinomiya R Sumida S Fujimoto S Okada A . Case report: Zolbetuximab-induced gastritis with protein-losing gastroenteropathy and hypogammaglobulinemia: a case implicating IgA vasculitis. Front Oncol. (2025) 15:1644263. doi: 10.3389/fonc.2025.1644263 40792275 PMC12336489

[40] Yamamoto K Owaki Y Nakayama I Sakamoto N Ishizaka M Kadota T . Characterization of early-onset gastritis during zolbetuximab-containing chemotherapy in CLDN18.2-positive gastric cancer. ESMO Open. (2025) 10:105805. doi: 10.1016/j.esmoop.2025.105805 41033281 PMC12514510

