# Supplementary material for: Integrating overall survival and health-related quality of life to prioritize first-line systemic therapies for unresectable advanced gastric cancer: a network meta-analysis
Source: Front Oncol. 2026 Sep 28;16:1910817. doi: 10.3389/fonc.2026.1910817 (PMC13654780; doi:10.3389/fonc.2026.1910817)
Supplement: Supplementary file 1 [file DataSheet1.docx]

**Supplementary material**

**Supplementary S1. Search strings**

**String search for PubMed**

((("Carcinoma, Gastric"[Mesh] OR "gastric adenocarcinoma"[tiab] OR GC[tiab] OR "gastric cancer"[tiab] OR "gastroesophageal junction adenocarcinoma"[tiab] OR GEJC[tiab] OR "gastroesophageal cancer"[tiab] OR "esophagogastric junction carcinoma"[tiab] OR "unresectable gastric cancer"[tiab] OR "advanced gastric cancer"[tiab] OR "metastatic gastric cancer"[tiab]))

AND

("platinum"[tiab] OR "fluoropyrimidine"[tiab] OR CAPOX[tiab] OR oxaliplatin[tiab] OR capecitabine[tiab] OR trastuzumab[tiab] OR "anti-HER2"[tiab] OR "HER2 inhibitor"[tiab] OR camrelizumab[tiab] OR tislelizumab[tiab] OR sintilimab[tiab] OR atezolizumab[tiab] OR pembrolizumab[tiab] OR durvalumab[tiab] OR tremelimumab[tiab] OR "immune checkpoint inhibitor*"[tiab] OR ICI[tiab] OR "PD-1"[tiab] OR "PD-L1"[tiab] OR "CTLA-4"[tiab] OR "tyrosine kinase inhibitor*"[tiab] OR TKI[tiab])

AND

("Quality of Life"[Mesh] OR "quality of life"[tiab] OR QoL[tiab] OR HRQoL[tiab] OR "patient reported outcome*"[tiab] OR PRO[tiab] OR PROs[tiab] OR "EORTC QLQ*"[tiab] OR "EORTC QLQ-STO22"[tiab] OR "EQ-5D"[tiab] OR "patient experience*"[tiab] OR "symptom burden"[tiab] OR "functional status"[tiab] OR "well-being"[tiab] OR "health status"[tiab])

AND

("Randomized Controlled Trial"[pt] OR "Controlled Clinical Trial"[pt] OR randomized[tiab] OR randomised[tiab] OR placebo[tiab] OR randomly[tiab] OR trial[tiab] OR "Clinical Trial, Phase III"[pt] OR "phase 3"[tiab] OR "phase III"[tiab]) AND

("first line"[tiab] OR "first-line"[tiab] OR frontline[tiab] OR "treatment naive"[tiab] OR "previously untreated"[tiab] OR "advanced"[tiab] OR "metastatic"[tiab] OR "unresectable"[tiab]))

**String search for Central**

("gastric adenocarcinoma" OR GC OR "gastric cancer" OR "gastroesophageal junction adenocarcinoma" OR GEJC OR "esophagogastric junction carcinoma" OR "unresectable gastric cancer" OR "advanced gastric cancer" OR "metastatic gastric cancer")

AND

(platinum OR fluoropyrimidine OR CAPOX OR oxaliplatin OR capecitabine OR trastuzumab OR "anti-HER2" OR "HER2 inhibitor" OR camrelizumab OR tislelizumab OR sintilimab OR atezolizumab OR pembrolizumab OR durvalumab OR tremelimumab OR "immune checkpoint inhibitor*" OR ICI OR "PD-1" OR "PD-L1" OR "CTLA-4" OR "tyrosine kinase inhibitor*" OR TKI)

AND

("quality of life" OR QoL OR HRQoL OR "patient reported outcome*" OR PRO OR PROs OR "EORTC QLQ*" OR "EORTC QLQ-STO22" OR "EQ-5D" OR "patient experience*" OR "symptom burden" OR "functional status" OR "well-being" OR "health status")

AND

(randomized OR randomised OR placebo OR randomly OR trial OR "phase 3" OR "phase III")

AND

("first line" OR "first-line" OR frontline OR "treatment naive" OR "previously untreated" OR advanced OR metastatic OR unresectable)

**String search for Scopus**

TITLE-ABS-KEY(("gastric adenocarcinoma" OR GC OR "gastric cancer" OR "gastroesophageal junction adenocarcinoma" OR GEJC OR "esophagogastric junction carcinoma" OR "unresectable gastric cancer" OR "advanced gastric cancer" OR "metastatic gastric cancer"))

AND

TITLE-ABS-KEY((platinum OR fluoropyrimidine OR oxaliplatin OR capecitabine OR CAPOX OR trastuzumab OR "anti-HER2" OR "HER2 inhibitor" OR camrelizumab OR tislelizumab OR sintilimab OR atezolizumab OR pembrolizumab OR durvalumab OR tremelimumab OR "immune checkpoint inhibitor*" OR ICI OR "PD-1" OR "PD-L1" OR "CTLA-4" OR "tyrosine kinase inhibitor*" OR TKI OR "anti-VEGF"))

AND

TITLE-ABS-KEY(("quality of life" OR QoL OR HRQoL OR "patient reported outcome*" OR PRO OR PROs OR "EORTC QLQ*" OR "EORTC QLQ-STO22" OR "EQ-5D" OR "patient experience*" OR "symptom burden" OR "functional status" OR "well-being" OR "health status"))

AND

TITLE-ABS-KEY((randomized OR randomised OR placebo OR randomly OR trial OR "phase 3" OR "phase III"))

AND

TITLE-ABS-KEY(("first line" OR "first-line" OR frontline OR "treatment naive" OR "previously untreated" OR advanced OR metastatic OR unresectable))

AND

DOCTYPE(ar OR cp)

**Supplementary S2. Data synthesis and analysis**

All network meta-analyses (NMAs) were performed in strict accordance with the PRISMA-NMA guidelines, the extended reporting standard for systematic reviews incorporating network meta-analysis. A Bayesian framework was adopted for the primary analysis of time to deterioration (TTD) of health-related quality of life (HR-QoL) domains and overall survival (OS). A frequentist random-effects NMA was subsequently conducted as sensitivity analysis to verify the robustness of primary findings. Meanwhile, network heterogeneity and loop inconsistency across the evidence network were assessed via the frequentist NMA approach.

Two ranking metrics were applied to hierarchically compare the efficacy of different interventions: the surface under the cumulative ranking curve (SUCRA) for Bayesian NMA and P-scores for frequentist NMA. The SUCRA value ranges from 0 to 1, where a value close to 1 indicates a higher probability that the corresponding treatment ranks the best among all evaluated regimens, and a value close to 0 denotes the worst performance. P-scores calculate the average probability that one intervention is superior to all competing treatments within the network.

An exploratory integrated analysis combining HR-QoL and OS outcomes was implemented using the minimum distance criterion method. This method integrates multiple clinical endpoints by minimising the Euclidean distance between observed outcomes and a theoretical optimal reference point (1,1), at which both HR-QoL and OS benefits are maximised. The Euclidean distance from each treatment to the optimal point (1,1) was calculated using the predefined formula as follows:

A smaller distance value represents a more favourable comprehensive performance across the two endpoints. This method generates a unified composite metric to balance HR-QoL and OS outcomes, enabling comprehensive hierarchical ranking of all included first-line treatments based on their overall clinical value.

Supplementary Figure 1. Network meta-analysis of comparisons of global health score/quality of life by EORTC-QLQ-C30. Node size is not proportional to sample size of included trials.

Supplementary Figure 2. Network meta-analysis of comparisons of physical functioning by EORTC-QLQ-C30. Node size is not proportional to sample size of included trials.

Supplementary Figure 3. Network meta-analysis of comparisons of nausea/vomiting by EORTC-QLQ-C30. Node size is not proportional to sample size of included trials.

Supplementary Figure 4. Network meta-analysis of comparisons of appetite loss by EORTC-QLQ-C30. Node size is not proportional to sample size of included trials.

Supplementary Figure 5. Network meta-analysis of comparisons of pain by EORTC-QLQ-STO22. Node size is not proportional to sample size of included trials.

Supplementary Figure 6. Network meta-analysis of comparisons of utility index by EQ-5D. Node size is not proportional to sample size of included trials.

## Supplementary Table 1. Characteristics of all studies

| Study name, year | Experimental arm | Control arm | HR-QoL questionnaire  (s) | Publication of HRQoL analysis |
| --- | --- | --- | --- | --- |
| RATIONALE-305,2025 | Tislelizumab | Chemotherapy | EORTC QLQ-C30; EORTC QLQ-STO22 | Main paper |
| KEYNOTE-811,2024 | Pembrolizumab plus Trastuzumab | Chemotherapy | EORTC QLQ-C30; EORTC QLQ-STO22 | Main paper |
| SPOTLIGHT, 2024 | Zolbetuximab | Chemotherapy | EORTC QLQ-C30; EORTC QLQ-OG25; EQ-5D | Main paper; Exploratory analysis |
| GLOW, 2024 | Zolbetuximab | Chemotherapy | EORTC QLQ-C30; EORTC QLQ-OG25; EQ-5D | Main paper |
| KEYNOTE-859,2023 | Pembrolizumab | Chemotherapy | EORTC QLQ-C30; EORTC QLQ-STO22 | Main paper |
| CheckMate 649,2021 | Nivolumab | Chemotherapy | FACT-Ga; EQ-5D | Main paper; Exploratory analysis |
| KEYNOTE-062,2019 | Pembrolizumab | Chemotherapy | EORTC QLQ-C30; EORTC QLQ-STO22 | Main paper; Exploratory analysis |
| ToGA,2010 | Trastuzumab | Chemotherapy | EORTC QLQ-C30; EORTC QLQ-STO22 | Main paper |
| RAINFALL,2019 | Ramucirumab | Chemotherapy | EORTC QLQ-C30 | Main paper |
| ATTRACTION-4,2022 | Nivolumab | Chemotherapy | FACT-Ga; EQ-5D | Main paper |

Abbreviation: HR-QoL, health related-quality of life. EORTC QLQ-C30, European Organisation for Research and Treatment of Cancer Quality of Life Questionnaire-Core 30; EORTC QLQ-STO22, European Organisation for Research and Treatment of Cancer Quality of Life Questionnaire-Stomach 22; EORTC QLQ-OG25, European Organisation for Research and Treatment of Cancer Quality of Life Questionnaire-Oesophago-Gastric 25; EQ-5D, EuroQol Five-Dimensional Questionnaire; FACT-Ga, Functional Assessment of Cancer – Gastric Adenocarcinoma.

Supplementary Table 2. Risk of bias assessment

| Study name, year/ risk of bias | Random sequence generation | Allocation concealment | Blinding (participants and personnel) | Blinding (outcome assessment) | Incomplete outcome data | Selective reporting |
| --- | --- | --- | --- | --- | --- | --- |
| RATIONALE-305,2025 | Low risk | Low risk | Low risk | Low risk | Low risk | Low risk |
| KEYNOTE-811,2024 | Low risk | Low risk | High risk | Low risk | Low risk | Low risk |
| SPOTLIGHT, 2024 | Low risk | Low risk | Low risk | Low risk | Low risk | Low risk |
| GLOW, 2024 | Low risk | Low risk | Low risk | Unclear risk | Low risk | Low risk |
| KEYNOTE-859,2023 | Low risk | Low risk | Low risk | Low risk | Unclear risk | Low risk |
| CheckMate 649,2021 | Low risk | Low risk | Low risk | Low risk | High risk | Low risk |
| KEYNOTE-062,2019 | Low risk | Low risk | Low risk | Low risk | Low risk | Low risk |
| ToGA,2010 | Low risk | Low risk | Low risk | Low risk | Low risk | Low risk |
| RAINFALL,2019 | Low risk | Low risk | High risk | Low risk | Low risk | High risk |
| ATTRACTION-4,2022 | Low risk | Low risk | Unclear risk | Low risk | Low risk | Low risk |

Supplementary Table 3. P-scores for EORTC-QLQ-C30 items calculated by frequentist network meta-analysis as sensitivity analysis.

| **HR-QoL Item** | **Treatment** | **P-score** |
| --- | --- | --- |
| **GHS/QoL** |  |  |
| GHS/QoL | Trastuzumab | 0.943 |
| GHS/QoL | Tislelizumab | 0.793 |
| GHS/QoL | Pembrolizumab | 0.584 |
| GHS/QoL | Zolbetuximab | 0.351 |
| GHS/QoL | Chemotherapy | 0.341 |
| GHS/QoL | Ramucirumab | 0.317 |
| GHS/QoL | Pembrolizumab plus Trastuzumab | 0.172 |
| **Physical functioning** |  |  |
| Physical functioning | Trastuzumab | 0.824 |
| Physical functioning | Tislelizumab | 0.665 |
| Physical functioning | chemotherapy | 0.663 |
| Physical functioning | Ramucirumab | 0.583 |
| Physical functioning | Pembrolizumab plus Trastuzumab | 0.155 |
| Physical functioning | Zolbetuximab | 0.110 |
| **Nausea/vomiting** |  |  |
| Nausea/vomiting | Pembrolizumab | 0.964 |
| Nausea/vomiting | Trastuzumab | 0.801 |
| Nausea/vomiting | Ramucirumab | 0.578 |
| Nausea/vomiting | Chemotherapy | 0.430 |
| Nausea/vomiting | Pembrolizumab plus Trastuzumab | 0.177 |
| Nausea/vomiting | Zolbetuximab | 0.051 |
| **Appetite loss** |  |  |
| Appetite loss | Trastuzumab | 0.901 |
| Appetite loss | Pembrolizumab | 0.707 |
| Appetite loss | Ramucirumab | 0.434 |
| Appetite loss | Chemotherapy | 0.358 |
| Appetite loss | Pembrolizumab plus Trastuzumab | 0.100 |
|  |  |  |

Abbreviation: GHS/QoL, global health status/quality of life.

Supplementary Table 4. P-scores for EORTC-QLQ-STO22/EQ-5D items calculated by frequentist network meta-analysis as sensitivity analysis.

| **HR-QoL Item** | **Treatment** | **P-score** |
| --- | --- | --- |
| **EORTC-QLQ-STO22** |  |  |
| **Pain** |  |  |
| Pain | Trastuzumab | 0.713 |
| Pain | Tislelizumab | 0.694 |
| Pain | Pembrolizumab | 0.430 |
| Pain | Pembrolizumab plus Trastuzumab | 0.370 |
| Pain | Chemotherapy | 0.292 |
| **EQ-5D** |  |  |
| **Utility index** |  |  |
| Utility index | Nivolumab | 0.988 |
| Utility index | Chemotherapy | 0.370 |
| Utility index | Zolbetuximab | 0.141 |
|  |  |  |

Abbreviations: EORTC-QLQ-STO22, the EORTC Quality of Life Questionnaire– Gastric Cancer Module; EQ-5D, EuroQol Five-Dimensional Questionnaire.

Supplementary Table 5. Ranking of the first-line systemic treatments for advanced gastric cancer according to their probability of being the best in overall survival.

| Treatment/ overall survival | SUCRA |
| --- | --- |
| Trastuzumab | 0.735 |
| Pembrolizumab | 0.686 |
| Zolbetuximab | 0.684 |
| Tislelizumab | 0.596 |
| Pembrolizumab plus Trastuzumab | 0.533 |
| Nivolumab | 0.491 |
| Ramucirumab | 0.187 |
| chemotherapy | 0.089 |

Supplementary Table 6. Ranking of the first-line systemic treatments according to the minimum distance criterion between OS and HR-QoL. Lower minimum distance criterion corresponds to improved net benefit across both OS and HR-QoL.

| EORTC-QLQ-C30 | | | | | | | |
| --- | --- | --- | --- | --- | --- | --- | --- |
| GHS/QoL | | Physical functioning | | Nausea/vomiting | | Appetite loss | |
| Treatment | MDC | Treatment | MDC | Treatment | MDC | Treatment | MDC |
| Trastuzumab | 0.287 | Trastuzumab | 0.301 | Pembrolizumab | 0.329 | Trastuzumab | 0.312 |
| Tislelizumab | 0.476 | Tislelizumab | 0.444 | Trastuzumab | 0.362 | Pembrolizumab | 0.448 |
| Pembrolizumab | 0.540 | chemotherapy | 1.079 | Ramucirumab | 0.920 | Ramucirumab | 0.984 |
| Zolbetuximab | 0.699 | Ramucirumab | 1.037 | chemotherapy | 1.067 | chemotherapy | 1.104 |
| chemotherapy | 1.113 | Pembrolizumab plus Trastuzumab | 0.798 | Pembrolizumab plus Trastuzumab | 0.901 | Pembrolizumab plus Trastuzumab | 0.954 |
| Ramucirumab | 1.043 | Zolbetuximab | 0.858 | Zolbetuximab | 0.949 |  |  |
| Pembrolizumab plus Trastuzumab | 0.908 |  |  |  |  |  |  |

Supplementary Table 7. network meta‑analysis excluding trials with post‑hoc HR‑QoL analyses

| Study | Questionnaire | Deterioration threshold | Requires confirmation at subsequent visit |
| --- | --- | --- | --- |
| RATIONALE‑305 | EORTC QLQ‑C30 / QLQ‑STO22 | ≥10‑point decrease from baseline | Yes |
| KEYNOTE‑811 | EORTC QLQ‑C30 / QLQ‑STO22 | ≥10‑point decrease from baseline | Yes |
| SPOTLIGHT | EORTC QLQ‑C30 / QLQ‑OG25 / EQ‑5D | ≥15‑point decrease from baseline | No |
| GLOW | EORTC QLQ‑C30 / QLQ‑OG25 / EQ‑5D | ≥15‑point decrease from baseline | No |
| KEYNOTE‑859 | EORTC QLQ‑C30 / QLQ‑STO22 | ≥10‑point decrease from baseline | Yes |
| CheckMate 649 | FACT‑Ga / EQ‑5D | ≥10‑point decrease from baseline | No |
| KEYNOTE‑062 | EORTC QLQ‑C30 / QLQ‑STO22 | ≥10‑point decrease from baseline | Yes |
| ToGA | EORTC QLQ‑C30 / QLQ‑STO22 | ≥10‑point decrease from baseline | Yes |
| RAINFALL | EORTC QLQ‑C30 | ≥10‑point decrease from baseline | No |
| ATTRACTION‑4 | FACT‑Ga / EQ‑5D | ≥10‑point decrease from baseline | No |

Supplementary Table 8. Heterogeneity and inconsistency statistics from frequentist network meta‑analysis for each HR‑QoL outcome

| Outcome domain | τ² | IF |
| --- | --- | --- |
| Global health status / Quality of life (GHS/QoL) | 0.042 | 0.31 |
| Physical functioning | 0.057 | 0.44 |
| Nausea/vomiting | 0.031 | 0.27 |
| Appetite loss | 0.064 | 0.52 |
| Pain (EORTC‑QLQ‑STO22) | 0.048 | 0.38 |
| Utility Index (EQ‑5D) | 0.025 | 0.21 |

Notes: This network has no closed loops. IF is merely for output reference.

Supplementary Table 9. SUCRA and P‑scores for sensitivity analysis excluding trials with post‑hoc HR‑QoL exploratory analyses

| GHS/QoL | | Physical functioning | | Nausea/vomiting | | Appetite loss | |
| --- | --- | --- | --- | --- | --- | --- | --- |
| Treatment | SUCRA | Treatment | SUCRA | Treatment | SUCRA | Treatment | SUCRA |
| Trastuzumab | 0.871 | Trastuzumab | 0.842 | Pembrolizumab | 0.887 | Trastuzumab | 0.816 |
| Tislelizumab | 0.733 | Tislelizumab | 0.801 | Trastuzumab | 0.741 | Pembrolizumab | 0.665 |
| Pembrolizumab | 0.547 | Chemotherapy | 0.434 | Ramucirumab | 0.552 | Ramucirumab | 0.431 |
| Zolbetuximab | 0.384 | Ramucirumab | 0.362 | Chemotherapy | 0.436 | Chemotherapy | 0.388 |
| Chemotherapy | 0.352 | Pembrolizumab plus Trastuzumab | 0.344 | Pembrolizumab plus Trastuzumab | 0.241 | Pembrolizumab plus Trastuzumab | 0.199 |
| Ramucirumab | 0.331 | Zolbetuximab | 0.217 | Zolbetuximab | 0.143 |  |  |
| Pembrolizumab plus Trastuzumab | 0.222 |  |  |  |  |  |  |

Supplementary Table 10. SUCRA and P‑scores for sensitivity analysis restricted to trials with TTD deterioration threshold ≥ 10‑points

| GHS/QoL | | Physical functioning | | Nausea/vomiting | | Appetite loss | |
| --- | --- | --- | --- | --- | --- | --- | --- |
| Treatment | SUCRA | Treatment | SUCRA | Treatment | SUCRA | Treatment | SUCRA |
| Trastuzumab | 0.863 | Trastuzumab | 0.848 | Pembrolizumab | 0.894 | Trastuzumab | 0.841 |
| Tislelizumab | 0.757 | Tislelizumab | 0.824 | Trastuzumab | 0.762 | Pembrolizumab | 0.693 |
| Pembrolizumab | 0.572 | Chemotherapy | 0.411 | Ramucirumab | 0.577 | Ramucirumab | 0.456 |
| Chemotherapy | 0.377 | Ramucirumab | 0.37 | Chemotherapy | 0.449 | Chemotherapy | 0.364 |
| Ramucirumab | 0.345 | Pembrolizumab plus Trastuzumab | 0.327 | Pembrolizumab plus Trastuzumab | 0.218 | Pembrolizumab plus Trastuzumab | 0.146 |
| Pembrolizumab plus Trastuzumab | 0.236 |  |  |  |  |  |  |

Supplementary Table 11. Minimum distance criterion (MDC) composite values under three alternative weighting schemes using global health status/quality of life (GHS/QoL) as representative health‑related quality‑of‑life domain

| weight analysis /Treatment | MDC (OS = 0.7 / HR‑QoL = 0.3) | MDC (OS = 0.3 / HR‑QoL = 0.7) |
| --- | --- | --- |
| Trastuzumab | 0.312 | 0.264 |
| Tislelizumab | 0.511 | 0.413 |
| Pembrolizumab | 0.582 | 0.507 |
| Zolbetuximab | 0.574 | 0.786 |
| chemotherapy | 0.841 | 0.942 |
| Ramucirumab | 0.976 | 1.081 |
| Pembrolizumab plus Trastuzumab | 1.145 | 1.092 |
